# Key factors influencing educational technology adoption in higher education: A systematic review

**DOI:** 10.1371/journal.pdig.0000764

**Published:** 2025-04-29

**Authors:** Jiyuan Feng, Bin Yu, Wee Hoe Tan, Zetian Dai, Zengkun Li

**Affiliations:** 1 Department of Education, Faculty of Social Sciences and Liberal Arts, UCSI University, Kuala Lumpur, Malaysia; 2 Pingdingshan university, Pingdingshan City, Henan Province, China; 3 School of Art, Suzhou University of Science and Technology, Suzhou, China; Fundación Progreso y Salud: Junta de Andalucia Consejeria de Salud y Familias Fundacion Progreso y Salud, SPAIN

## Abstract

In the current globalised educational environment, higher education increasingly relied on educational technology to enhance teaching and learning outcomes. Therefore, exploring the decisive factors influencing the adoption of educational technology was crucial for its successful implementation. This paper employed a systematic review using the PRISMA method to investigate four key dimensions affecting educational technology adoption: Performance Expectancy, Effort Expectancy, Social Influence, and Facilitating Conditions. Through a thorough examination of relevant literature, this review deepened the understanding of the core factors influencing the adoption process. A total of 1,891 studies related to educational technology adoption, published between 2015 and 2024, were initially identified, with 39 studies remaining after careful selection for analysis. The classification analysis revealed that all articles were categorised under the four themes: Performance Expectancy (8 articles), Effort Expectancy (18 articles), Social Influence (5 articles), and Facilitating Conditions (8 articles). This review provided valuable insights for higher education institutions aiming to enhance educational quality through the adoption of advanced educational technologies, and it also made a significant contribution to the existing academic literature. However, the interactions between the four dimensions warrant further exploration.

## Author summary

The adoption of educational technology in higher education has become a crucial driver for enhancing teaching effectiveness and learning outcomes. This study systematically reviewed literature from 2015 to 2024 to identify key factors influencing educational technology adoption. Using the PRISMA framework, 39 studies were selected and analyzed under four dimensions: Performance Expectancy, Effort Expectancy, Social Influence, and Facilitating Conditions. Findings revealed that Performance Expectancy, or the perceived benefits of technology in improving learning, played a pivotal role in adoption. Effort Expectancy, related to ease of use and accessibility, significantly influenced engagement. Social Influence, including peer, institutional, and cultural factors, shaped attitudes toward technology adoption. Lastly, Facilitating Conditions, such as infrastructure and technical support, were critical enablers of successful implementation. While educational technology offers transformative potential, challenges such as digital inequality, institutional resistance, and inadequate support structures persist. This review provides valuable insights for educators, policymakers, and technology developers to design strategies that optimize technology integration in higher education. Future research should explore the interplay between these dimensions and the long-term impact of digital transformation in academia.

## Introduction

In the context of rapid development in high-tech technologies, learning, using, evaluating, and updating educational technology had become essential conditions for higher education institutions to maintain teaching effectiveness and achieve educational goals [1]. The Education 4.0 era had begun, a concept derived from Industry 4.0, which emphasized personalised learning, technological integration, and data-driven decision-making processes. In the Education 4.0 era, the specific requirements facing educational systems included enhancing adaptability, interactivity, and sustainability in education while also demanding that educators not only impart knowledge but also stimulate innovative thinking and problem-solving abilities. This era particularly underscored the use of the Internet and other educational technologies, such as online learning platforms and virtual reality, to enhance the efficiency of learning experiences and educational outcomes [2]. The utilisation of modern educational technology played a crucial role in higher education, not only transforming the methods of teaching and learning but also driving the continuous improvement and innovation of educational quality [3].

Educational technology began to emerge in the mid-20th century with the increasing use of television and other media in education, which also saw the development of related terms and concepts. In the 1950s and 1960s, multimedia and television were introduced into classrooms for teaching purposes. By the 1970s, with the advent of computer technology, computer-assisted instruction became possible. The 1990s witnessed the widespread adoption of the Internet, marking a new chapter in online education and popularising distance education [4]. Entering the 21st century, the widespread use of mobile devices such as smartphones and tablets, along with the recent application of artificial intelligence and big data technologies, had further propelled the evolution of educational technology towards mobile learning and smart education [5].

Educational technology, commonly referred to as “Information and Communication Technology (ICT) in education,” pertained to the practice of using computer hardware, software, and other technological media to support learning and teaching [6]. The use of educational technology could enhanced educational efficiency, improved the learning experience, and cater to the diverse learning needs of students [7]. In higher education, the application of educational technology ranged from online course management systems to interactive learning tools and virtual laboratories, with the primary goals of enhancing teaching interactivity, personalising learning paths, and ensuring the real-time accuracy of student performance assessment [8]. Educational technology has sparked a revolution in higher education, not only altering traditional teaching models but also providing greater flexibility and accessibility for both students and teachers, thereby improving the overall quality and effectiveness of instruction.

Despite the numerous advantages of educational technology, socioeconomic barriers significantly impacted its adoption and effectiveness in higher education worldwide. Financial constraints, digital divides, and disparities in technological infrastructure created substantial gaps in access and usage, particularly in developing countries and economically disadvantaged communities. Studies showed that students from low-income backgrounds often lacked access to high-speed internet, personal computing devices, and digital literacy training, which hindered their ability to fully engage with online learning environments [9]. Furthermore, even in technologically advanced regions, socioeconomic disparities affected students’ access to supplementary learning resources, creating an uneven playing field in digital education [10]. Research highlighted that while universities in wealthier nations could afford to implement advanced educational technologies, institutions in resource-limited settings struggled with outdated infrastructure and insufficient technical support, reducing the effectiveness of technology-enhanced learning initiatives [11]. Addressing these socioeconomic barriers was crucial for ensuring the equitable adoption of educational technology and maximising its potential to enhance learning outcomes for all students [12].

The era of high technology and the internet has endowed the field of education with greater possibilities. Higher education institutions utilised educational technology primarily in course delivery, student support services, curriculum development, as well as the assessment and monitoring of student performance [13]. The initial implementation of educational technology in the educational sector could be traced back to the early 1990s with the rise of online learning and distance education, whereas its widespread adoption in higher education began in the early 21st century [14]. As broadband internet and mobile technologies became more prevalent, their impact grew increasingly profound [15]. The introduction of educational technology has increased the proportion of online courses; by 2019, over 30% of higher education students had enrolled in at least one online course [16]. Additionally, educational technology has promoted the development of flipped classrooms and blended learning models, which had been proven to enhance student engagement and academic performance [17].

Educational technology played a crucial role in higher education, primarily by introducing innovative teaching tools and platforms to enhance teaching interactivity and learning efficiency [18]. Nevertheless, in practice, educational technology had not always achieved the anticipated educational goals, often being constrained by a lack of complementary educational strategies and sufficient technical support [19]. Furthermore, higher education institutions faced various challenges in implementing educational technology, such as financial limitations, outdated technological updates, and a shortage of skilled technical personnel [20,21]. These issues further exacerbated the resistance and obstacles encountered by teachers and students when adopting new technologies, resulting in incompatibilities between technology and traditional teaching methods [22]. Moreover, the existing literature on this topic provided limited findings and lacked satisfactory evidence regarding the factors that determine the use of educational technology [23]. This highlighted the necessity of investigating the factors influencing the adoption of educational technology to achieve satisfactory teaching outcomes [24].

Therefore, this study employed a systematic review approach, using the Unified Theory of Acceptance and Use of Technology (UTAUT) as the framework to thoroughly examine the various factors that might have affected the adoption process of educational technology in the field of higher education [25]. The UTAUT theory was proposed by Venkatesh, Morris, Davis, and Davis in 2003, with their research findings published in MIS Quarterly. UTAUT integrates eight existing technology acceptance models and theories, including the Technology Acceptance Model (TAM), the Theory of Planned Behavior (TPB), the extended Technology Acceptance Model (TAM2), the Motivational Model of PC Utilization (MPCU), the Innovation Diffusion Theory (IDT), the Social Cognitive Theory (SCT), the Model of Organizational Behavior, and the Social Influence Theory (SI). By integrating these models, UTAUT provided a unified theoretical framework to explain the broad factors influencing individual acceptance and use of technology [26]. It identified four core constructs that determine the intention to accept and use technology: Performance Expectancy, Effort Expectancy, Social Influence, and Facilitating Conditions [27]. Performance Expectancy refers to the individual’s belief that using technology would enhance their work or learning performance [28]. Effort Expectancy referred to the perceived ease of use of the technology. Social Influence referred to the perceived influence of the opinions of significant others, such as family, friends, and colleagues, on the individual’s decision to use the technology. Facilitating Conditions referred to the perceived availability of organisational and technical infrastructure that supported the use of technology [29].

This research held significant value for researchers and practitioners in the fields of education, educational technology, and data analysis, as it provided a comprehensive overview of the existing literature on the relationship between PESF factors and technology adoption (i.e., educational technology) within the educational domain. The findings of this study could have assisted researchers in higher education in locating relevant literature on the adoption of educational technology, as well as contributed to a better understanding of the factors influencing the adoption of educational technology, thereby aiding in the more effective application of educational technology at the educational level.

This study began by outlining the research methodology, providing a detailed explanation of the systematic review mapping process, and presenting the research findings based on a classification framework. The research then discussed the relevant literature on the adoption of educational technology in higher education from the four dimensions of Performance Expectancy, Effort Expectancy, Social Influence, and Facilitating Conditions. The study concludes with a summary of the research findings, highlighting the study’s limitations and suggesting directions for future research.

## Research methodology

A Systematic Literature Review (SLR) is a scientific research method used to answer specific research questions by systematically searching, evaluating, and integrating existing research materials. SLR emphasised the use of explicit, replicable methods to identify, select, and critically analyse relevant studies, thereby reducing bias and providing reliable conclusions [30]. In this study, SLR was employed as an appropriate method to thoroughly investigate the literature on factors influencing the adoption of educational technology in higher education. Following the standard SLR process, the study defined research questions, sets inclusion and exclusion criteria, developed a search strategy, searched the literature, screened the literature, conducted quality assessments, extracted and synthesised data, and reported and disseminated the results [31]. This analysis used the Preferred Reporting Items for Systematic Reviews and Meta-Analyses (PRISMA) method, a reporting standard for conducting systematic literature reviews. Generally, this method helped authors evaluate and review the quality and rigour of the literature and provided relevant and necessary details [32]. According to the method proposed by Ali et al., the SLR analysis was conducted in three distinct stages [33].

### Planning stage

In the field of education, the adoption of educational technology had become a common option, making educational technology increasingly important in education [34]. To date, there had not been a systematic review that comprehensively synthesised these research findings and provided an in-depth analysis of the academic aspects of this topic.

The first step in planning the analysis was to develop a research protocol. This protocol clarified the research objectives, key questions, and expected outcomes, ensuring that the entire review process was orderly and yielded high-standard results. The classification framework of this study included four dimensions: Performance Expectancy, Effort Expectancy, Social Influence, and Facilitating Conditions, with several factors grouped according to the selected review results [35]. This framework provided a structured approach to analysing the relevant literature on factors influencing the adoption of educational technology in higher education.

Secondly, concerning the research questions of this review, these questions served as a guide throughout the review process, including literature retrieval, study selection, and synthesis of research findings. Therefore, for this review, the research question was as follows: What factors significantly influence the adoption of educational technology in higher education institutions?

Subsequently, the design of the literature search strategy was undertaken, including the selection of appropriate databases, the formulation of keywords, and the setting of search parameters. These steps were intended to collect as comprehensively as possible the relevant literature on the adoption of educational technology in higher education [36]. The research team established eligibility criteria for selection, as shown in Table 1. The inclusion/exclusion criteria ensure that the literature was relevant to the research question. Additionally, this method ensured that the evaluation results were more accurate, objective, and meaningful while minimising the risk of bias and maintaining the integrity of the systematic review.

**Table 1:** Research selection criteria.

Criterion	Inclusion	Exclusion
**Language**	English	Non-English
**Timeline**	2015-2024	<2015
**Literature type**	Journal (only research articles)	Journal (book chapter, conference proceeding)

This study employed WoS, Scopus, and Emerald as electronic databases to conduct extensive automated searches and a thorough manual review of selected articles. The database searched for this review included databases related to education, information technology, and social sciences. Since there was no absolutely perfect and comprehensive database, the review encompassed fields such as economics, information technology, education, social sciences, literature, medicine, and general science [37]. The following was elaborated into three subsections: identification, screening, and eligibility assessment [38].

### Implementation phase

#### Identification.

The systematic review process was divided into three stages, each responsible for selecting the most relevant papers for this study. The first step to be completed was identification, which involved searching for relevant literature across a wide range of databases and resources. This typically included defining keywords, search criteria, and the databases to be included. Researchers ensured that the search strategy was both comprehensive and targeted to capture all relevant studies [39]. Therefore, after identifying all relevant keywords, a keyword search was conducted, and search strings were developed for the WoS, Scopus, and Emerald databases (see Table 2). The specific content in Table 2 represented the search strings used when searching the databases. In the first step of the systematic review, this study successfully retrieved 1,891 journal articles from the databases.

**Table 2:** The search strings.

Databases	Search strings
**SCOPUS**	(TITLE-ABS-KEY (educational AND technology) AND TITLE-ABS-KEY (determining AND factor) AND TITLE-ABS-KEY (higher AND education)) AND (LIMIT-TO (PUBYEAR, 2015) OR LIMIT-TO (PUBYEAR, 2016) OR LIMIT-TO (PUBYEAR, 2017) OR LIMIT-TO (PUBYEAR, 2018) OR LIMIT-TO (PUBYEAR, 2019) OR LIMIT-TO (PUBYEAR, 2020) OR LIMIT-TO (PUBYEAR, 2021) OR LIMIT-TO (PUBYEAR, 2022) OR LIMIT-TO (PUBYEAR, 2023) OR LIMIT-TO (PUBYEAR, 2024)) AND (LIMIT-TO (DOCTYPE, “ar”)) AND (LIMIT-TO (LANGUAGE, “English”))
**Web of Science**	educational technology (All Fields) and determining factor (All Fields) and field of higher education (All Fields) and 2024 or 2023 or 2022 or 2021 or 2020 or 2019 or 2018 or 2017 or 2016 or 2015 (Publication Years) and English (Languages) and Article (Document Types)
**Emerald**	(content-type: article) AND (Determining the factors)AND(educational technology adoption in the field of higher education)AND2024 or 2023 or 2022 or 2021 or 2020 or 2019 or 2018 or 2017 or 2016 or 2015 (Publication Years) AND English (Languages)

#### Screening.

At this stage, the focus of the study was on research articles, excluding other forms of publications, to ensure research depth. Items such as systematic reviews, commentaries, monographs, book chapters, and conference proceedings were excluded. Additionally, the study refined the selection to include only papers written in English to ensure universality and comprehensibility. To ensure the timeliness of the research, a timeframe from 2015 to 2024 was established (see Table 2). A comprehensive review of all article titles and abstracts was conducted, and studies that clearly did not meet the inclusion criteria were excluded. At this stage, duplicate papers were also filtered out. Based on these rigorous screening criteria, 1,327 papers that did not meet the requirements were excluded, allowing for a deeper eligibility assessment of the remaining literature. This process ensured the selection of the most relevant and up-to-date research on the topic.

#### Eligibility.

The purpose of the eligibility assessment stage was to further examine the articles that passed the previous screening phase to ensure that they meet the specific requirements and standards of the study. This stage was critical in the literature review process, as it represented the final checkpoint for determining the quality and relevance of the reviewed literature. The study assessed the suitability of each article by thoroughly reviewing key aspects such as research design, study samples, interventions, and research outcomes, excluding articles with questionable research quality, incomplete data, or irrelevant results [40]. During the eligibility assessment stage, 521 articles were prepared for review. In this stage, a comprehensive review of the titles and main content of all articles was conducted to ensure they met the inclusion criteria and aligned with the current research objectives of the study. As a result, 482 reports were excluded due to a lack of clear relevance to the research objectives. Ultimately, 39 articles were deemed suitable for inclusion and review.

### Summarization stage

**Fig 1:**
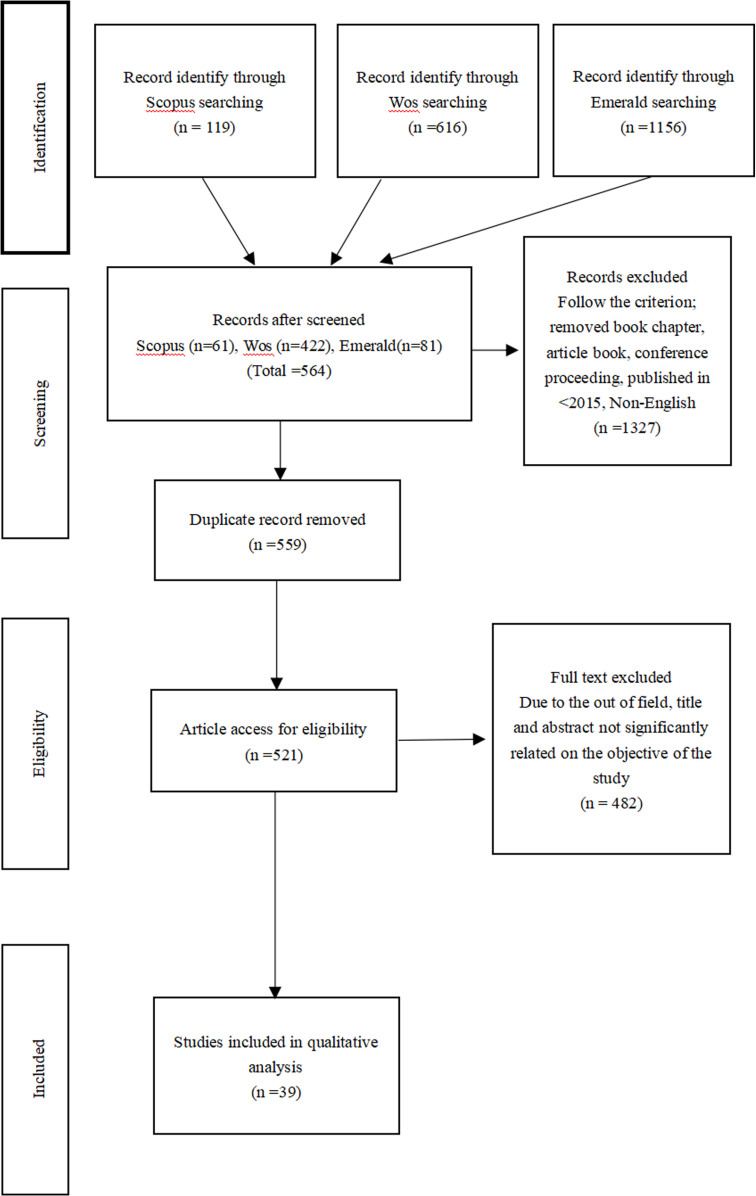
Flow diagram of the proposed search study [41].

During the initial automated search process using keywords, a total of 1,891 articles were generated. Filters were then applied to the retrieved articles, which eliminated 1,327 entries, resulting in 564 articles. Afterwards, duplicate entries were removed, yielding 559 articles. These remaining articles were manually reviewed by reading their titles and abstracts to determine their relevance, with a particular focus on those closely related to the research topic. The articles that passed this review underwent further screening based on their full content, and 521 articles were categorised as relevant to the study’s theme. These articles were then subjected to a final manual review. Ultimately, based on the quality assessment criteria previously described, 39 articles were selected for analysis (Fig 1). During the data extraction process, particular attention was paid to each article’s main research objectives, methods, results, and specific discussions on factors influencing the adoption of educational technology. By synthesising this data, the study aimed to identify the factors that influence the adoption of educational technology in higher education. This research will also identified and discussed any significant trends, challenges, and gaps for future research discovered in the conducted surveys. These findings, based on existing studies, provided valuable insights for researchers and educators, aiding in the understanding and advancement of educational technology. Furthermore, the analysis results informed the direction of future research.

## Results

### Common attributes of selected articles

#### Temporal distribution of selected studies.

**Fig 2:**
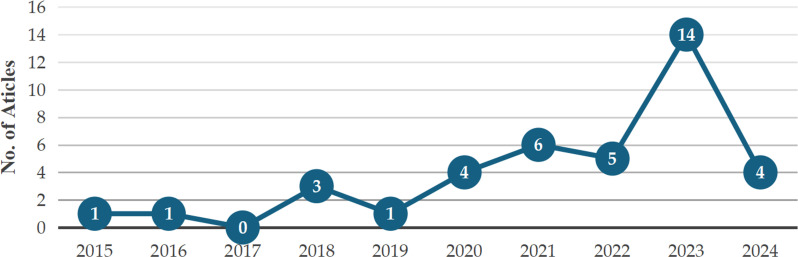
Publications by years from 2015 to 2024.

Fig 2 illustrated the temporal distribution of the articles selected for this review, covering the period from 2015 to 2024. No articles were published in 2017, while three articles were published in 2018. In 2015, 2016, and 2019, one article was published each year. Subsequently, the number of articles gradually increased, with four published in 2020, six in 2021, and nine in 2022. The year 2023 saw the highest number of publications, with a total of 14 articles. As of August 2024, four articles had been published. These results indicated a sustained increase in research interest in the adoption of educational technology over the years, with 2023 marking a peak in academic activity within this field. This trend clearly reflected the growing adoption of technology in education and the increasing focus on exploring the changes it brought to the field.

#### Geographic distribution of selected studies.

**Fig 3:**
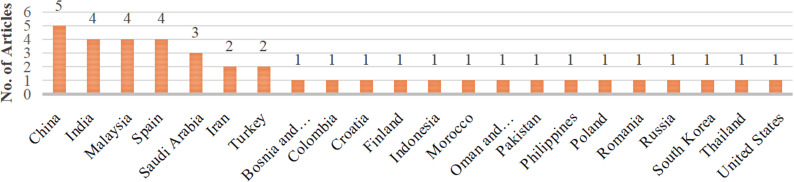
Distribution of articles by country.

The 39 studies selected for this review encompassed at least 22 different countries, as shown in Fig 3. China ranked first with five studies, followed by Malaysia, India, and Spain with four studies each, and Saudi Arabia with three studies. Additionally, Iran and Turkey each had two studies, while the remaining countries were represented by one study each. Overall, the majority of the studies in this review were conducted in Asia, where this research topic had a significant presence.

### Research classification framework

**Fig 4:**
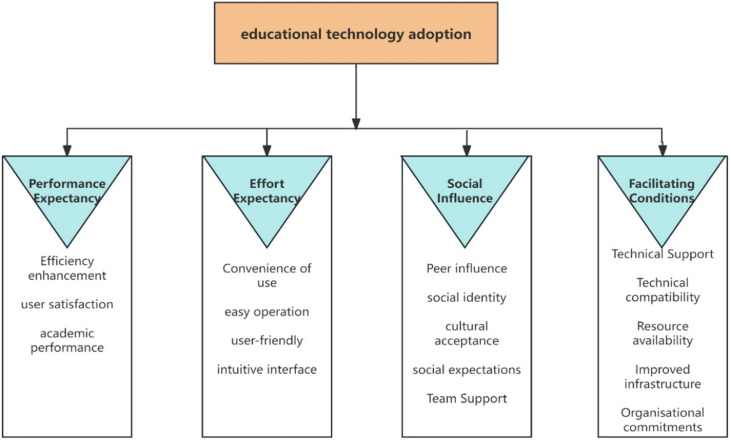
Research classification framework.

This study conducted a comprehensive review of academic articles related to factors that may influence the adoption of educational technology in higher education. To facilitate the examination, a classification framework was adopted, as illustrated in Fig 4. This framework includes an analysis of four key aspects: Performance Expectancy, Effort Expectancy, Social Influence, and Facilitating Conditions. A more detailed breakdown of the 39 selected articles was provided in the accompanying Table 3.

**Table 3:** The research article’s findings based on the proposed search criterion. Performance Expectancy: 1, Effort Expectancy: 2, Social Influence: 3, Facilitating Conditions: 4.

NO	Author	Journal	Title	Scopus	WoS	emerald	Remarks
1	Ashraf et al.	(2023) International Journal of Environmental Research and Public Health	Acceptance of Smart Technologies in Blended Learning: Perspectives of Chinese Medical Students	✓			1
2	Pérez-Pérez et al.	(2020) Journal of Further and Higher Education	An analysis of factors affecting students′ perceptions of learning outcomes with Moodle	✓	✓		2
3	Raza et al.	(2018) International Journal of Engineering and Technology (UAE)	Analysis determinants of social media acceptance in higher educational institutes of Pakistan	✓			3
4	Aznar-Díaz et al.	(2020) Computers and Education	Analysis of the determining factors of good teaching practices of mobile learning at the Spanish University. An explanatory model	✓	✓		4
5	Bessadok, Adel	(2022) Education and Information Technologies	Analyzing student aspirations factors affecting e-learning system success using a structural equation model	✓	✓		1
6	Dai et al.	(2021) IEEE Access	Assessment of Smart Learning Environments in Higher Educational Institutions: A Study Using AHP-FCE and GA-BP Methods	✓	✓		4
7	Adams et al.	(2020) Malaysian Journal of Learning and Instruction	Blended learning engagement in higher education institutions: A differential item functioning analysis of students’ backgrounds	✓	✓		3
8	Diteeyont, Watsatree; Heng-Yu, Ku	(2023) Smart Learning Environments	Competency levels and influential factors of college student’ mobile learning readiness in Thailand	✓	✓		2
9	Prasetyo et al.	(2021) Healthcare (Switzerland)	Determining Factors Affecting the Acceptance of Medical Education eLearning Platforms during the COVID-19 Pandemic in the Philippines: UTAUT2 Approach	✓	✓		1
10	Chinchilla, Leidys del Carmen Contreras	(2018) International Journal of Technologies in Learning	Determining factors in Web 2.0 adoption by university students	✓			2
11	Adi et al.	(2023) Sport TK	Digital literacy of physical education teachers in the 5.0 era	✓	✓		2
12	Tuta, Jadranko; Luić, Ljerka	(2024) Education Sciences	D-Learning: An Experimental Approach to Determining Student Learning Outcomes Using Augmented Reality (AR) Technology	✓	✓		1
13	Kim et al.	(2023) Applied Sciences	Effect of Block-Based Python Programming Environment on Programming Learning	✓	✓		2
14	Gupta et al.	(2023) Global Knowledge, Memory and Communication	E-leadership and virtual communication adoption by educators: an UTAUT3 model perspective	✓	✓	✓	4
15	Suparman et al.	(2023) Comunicar	English learners’ intentions to adopt online learning post-pandemic: Ease precedes usefulness	✓	✓		2
16	Almogren et al.	(2024) Heliyon	Exploring factors influencing the acceptance of ChatGPT in higher education: A smart education perspective	✓	✓		2
17	Zayim, Nese; Ozel, Deniz	(2015) CIN - Computers Informatics Nursing	Factors affecting nursing students’ readiness and perceptions toward the use of mobile technologies for learning	✓	✓		2
18	Almaiah et al.	(2022) Electronics (Switzerland)	Factors Affecting the Adoption of Digital Information Technologies in Higher Education: An Empirical Study	✓	✓		2
19	Ejdys, Joanna	(2021) WSEAS Transactions on Business and Economics	Factors affecting the adoption of e-learning at university level	✓			1
20	Rabiei et al.	(2023) Health Education and Health Promotion	Factors Associated with the Use of E-Learning among Medical University Students; An Application of Technology Acceptance Model	✓			2
21	Cazan, Maria; Maican, Catalin-Ioan	(2023) Comunicar	Factors determining the use of e-learning and teaching satisfaction	✓	✓		2
22	Nikou, Shahrokh; Maslov, Ilia	(2023) International Journal of Educational Management	Finnish university students’ satisfaction with e-learning outcomes during the COVID-19 pandemic	✓	✓	✓	2
23	Al-Emran et al.	(2016) Computers in Human Behavior	Investigating attitudes towards the use of mobile learning in higher education	✓	✓		2
24	Huang et al.	(2022) Interactive Learning Environments	Investigating the antecedents of university students’ perceived ease of using the Internet for learning	✓	✓		2
25	Aliaño, Ángel Mojarro et al.	(2019) Journal of New Approaches in Educational Research	Mobile Learning in University Contexts Based on the Unified Theory of Acceptance and Use of Technology (UTAUT)	✓	✓		3
26	Gulyaeva et al.	(2020) Universidad y Sociedad	New information technologies as the basis for improving the quality of higher professional education	✓	✓		4
27	Ho et al.	(2021) PLoS ONE	Predicting student satisfaction of emergency remote learning in higher education during COVID-19 using machine learning techniques	✓	✓		1
28	Becirovic, Senad; Dervic, Mersad	(2023) Electronic Journal of Information Systems in Developing Countries	Students’ perspectives of digital transformation of higher education in Bosnia and Herzegovina	✓	✓		3
29	Shaikh et al.	(2024) On the Horizon	Students’ e-learning acceptance: Empirical evidence from higher learning institutions	✓	✓		2
30	Love et al.	(2018) Medical Science Educator	Supporting the Professional Identity of Medical Science Educators: Understanding Faculty Motivations for Quality Improvement in Teaching	✓			1
31	Unal, Erhan; Uzun, Ahmet Murat	(2021) British Journal of Educational Technology	Understanding university students’ behavioral intention to use Edmodo through the lens of an extended technology acceptance model	✓	✓		2
32	Rosli et al.	(2024) Data in Brief	Unlocking insights: A comprehensive dataset analysis on the acceptance of computational thinking skills among undergraduate university students through the lens of extended technology acceptance model, HTMT, covariance-based SEM, and SmartPLS	✓	✓		2
33	Romero-Rodríguez et al.	(2023) Journal of New Approaches in Educational Research	Use of ChatGPT at University as a Tool for Complex Thinking: Students’ Perceived Usefulness	✓	✓		3
34	Weng, Shaobin; Qin, Yuanyuan	(2023) Environmental Science and Pollution Research	Which qualities should built environment possess to ensure satisfaction of higher-education students with remote education during pandemics?	✓	✓		4
35	Karimian et al.	(2023) BMC Medical Education	Which virtual education methods do e-students prefer? Design and validation of Virtual Education Preferences Questionnaire (VEPQ)	✓	✓		4
36	Ravichandran, Bargavi Shanmugam, Kavitha	(2023) Management Matters	Adoption of EdTech products among college students: a conceptual study			✓	4
37	Alami et al.	(2022) Arab Gulf Journal of Scientific Research	Students’ adoption of e-learning: evidence from a Moroccan business school in the COVID-19 era	✓		✓	2
38	Dubey, Pushkar; Sahu, Kailash Kumar	(2021) Journal of Research in Innovative Teaching and Learning	Students’ perceived benefits, adoption intention and satisfaction to technology-enhanced learning: examining the relationships	✓		✓	1
39	Mukherjee, Mrinal; Maity, Chanchal	(2022) Asian Association of Open Universities Journal	Emergency remote learning (ERL) in the COVID-19 era: perceived experience of Indian learners of higher education	✓		✓	4

## Discussion

With the rapid development of technology in the educational sector, the adoption of educational technology became a crucial means of enhancing learning efficiency and quality. The application of various educational tools and platforms redefined traditional teaching and learning methods, making them more flexible and interactive. However, the successful adoption of educational technology was not without challenges, as it involved multiple factors at the technological, social, and individual levels. Understanding these factors was essential for designing and implementing effective educational technology strategies.

### Performance expectancy

In today’s educational environment, performance expectancy became a key driver for higher education institutions to adopt new technologies. As educational technology rapidly evolved, its potential to enhance teaching and learning efficiency was increasingly recognised. This expectation motivated educators and students to explore how technology could enhance the learning experience to achieve better educational outcomes.

Firstly, the effective adoption of intelligent technology in blended learning environments relied on appropriate social contexts and organisational support, while individual student behaviour was significantly influenced by performance expectancy, effort expectancy, social influence, and hedonic motivation [42]. Additionally, during the COVID-19 pandemic, performance expectancy emerged as the primary factor influencing the acceptance of e-learning platforms among medical students in the Philippines [43]. Equally important, factors such as student motivation, expectations, and enjoyment played a critical role in the successful adoption of e-learning systems, particularly in the intention to use and perceived usefulness of these systems [44]. Large-scale data analysis revealed that students’ perceived usefulness served as a significant mediator and moderator in the relationship between their intention to adopt technology-enhanced learning (TEL) and their overall satisfaction [45].

In practical research, scenario-based experiments, game-based experiments, and research experiments demonstrated that actively using various forms of augmented reality in the learning process could significantly improve students’ knowledge levels, confirming that the application of augmented reality technology in teaching can notably enhanced learning outcomes, with student initiative being considered an important factor [46]. Furthermore, the level of effort exerted by teachers, the appropriateness of assessment methods following the use of educational technology, and perceptions of the effective delivery of online learning had a significant impact on student satisfaction, which in turn influenced the use of educational technology [47]. Moreover, intrinsic motivation was a key factor driving teachers to pursue improvements in teaching quality and performance, with student engagement, the use of instructional technology, and demonstration or guidance skills being the primary motivators. These factors informed managers, educational leaders, teacher developers, and educators about what supported the effectiveness and professional identity of higher education faculty in the use of educational technology [48]. Research on the adoption of e-learning systems by Polish university students revealed that challenges in adopting educational technology included perceived ease of use, availability of facilitating conditions, and adequacy of technical support. Specifically, perceived usefulness played a critical role in shaping students’ attitudes toward educational technology [49].

In summary, performance expectancy involved not only the technical performance but also how educators and learners perceived the potential benefits these technologies offered. Educational institutions need to continually assess and adapt these tools to ensure they effectively enhanced the quality of teaching and learning.

### Effort expectancy

In the current higher education environment, the adoption and implementation of technology became pivotal factors in transforming teaching and learning methods. Particularly within the dimension of effort expectancy, ease of use played a pivotal role in determining sustained engagement with educational technology. A deeper understanding of how usability impacted students and educators could have enhanced its effectiveness and long-term adoption in academic settings.

Firstly, research on Learning Management Systems (LMS) revealed that information quality was a core predictor of student satisfaction, with the effectiveness of technology communication playing a crucial role in enhancing students’ perceptions of learning outcomes [50]. Additionally, a comparative review of existing theories and case studies showed that ease of use, flexibility, interactivity, availability of educational materials, and accessibility of distributed content were key factors influencing students’ adoption of Web 2.0 technologies [51].

Moreover, case studies from different countries emphasised the impact of environmental factors on technology acceptance. In Thailand, factors such as technological readiness, self-directed learning, and netiquette significantly influenced students’ readiness for mobile learning, with learners’ technological preparedness being the most significant factor affecting their use of learning technology [52]. Similarly, research in Oman and the UAE highlighted that students’ and educators’ attitudes towards technology were decisive in their readiness to adopt digital learning tools [53]. In China, computer self-efficacy, perceived enjoyment, and perceived external control significantly influenced the perceived ease of use of technology, which in turn shaped students’ attitudes towards using online platforms for learning [54]. A study on Moroccan business school students during the COVID-19 pandemic showed that student satisfaction, perceived ease of use, and perceived usefulness were key determinants of e-learning acceptance [55].

Regarding specific applications of technology, the quality and accessibility of digital learning platforms, as well as course design, directly impacted students’ satisfaction and engagement with e-learning tools [56]. Mobile technology adoption by university students was also closely tied to perceived ease of use, perceived usefulness, personal innovativeness, and self-management in learning [57], while their intention to use digital platforms depended largely on their perceptions of usability and utility [58]. Notably, research on online English language learning tools demonstrated that perceived ease of use had a greater impact than perceived usefulness on students’ attitudes and intentions, further highlighting the role of usability [59]. Similarly, studies on AI-driven chatbots, such as ChatGPT, reinforced that perceived ease of use and usefulness were key factors in user acceptance [60].

Research on the adoption of digital information technology in higher education indicated that users’ perceptions of the value of educational digital information depended on external conditions, particularly the level of technological preparedness, which was crucial for determining perceived ease of use [61]. In medical universities, studies on the use of educational technology revealed that students’ age, history, and academic level were significantly associated with their willingness to engage in e-learning, suggesting that students from different backgrounds had varying effort expectancies regarding e-learning. Attitudes, perceived usefulness, and ease of use were the strongest predictors of the willingness to use e-learning [62]. In the face of uncertainty, technological pressure and self-efficacy had been identified as key factors determining students’ adoption of e-learning tools [63]. In physical education, teachers’ digital literacy, particularly their level of technological proficiency, significantly influenced their use of technology in teaching. Younger teachers with better technological proficiency were more inclined to use educational technology [64]. In programming education, students’ perceptions of programming at different learning stages were influenced by teaching methods, with both secondary and university students relying to varying degrees on the perceived difficulty of this computer technology [65].

Finally, studies on business students’ adoption of e-learning systems underscored that online collaborative learning and technological self-efficacy were among the most influential factors, with collaborative learning having the strongest impact [66]. Likewise, research on undergraduate students’ acceptance of computational thinking provided insights into the diverse requirements different learner groups had for technology adoption, particularly in terms of usability and perceived usefulness [67].

Higher education institutions needed to recognise that improving the ease of use and accessibility of technology was key to enhancing user acceptance and satisfaction. As technology played an increasingly important role in education, continuously optimizing the design and functionality of these tools to meet the diverse needs of users would have been central to the successful implementation of educational technology in the future.

### Social influence

As technology continues to penetrate deeper into education, social influence played a crucial role in the acceptance and adoption of educational technology. Different cultural backgrounds and social environments could have significantly impacted the attitudes and willingness of students and teachers to use new technologies. This influence was particularly prominent in higher education worldwide, where social factors become key drivers or obstacles to the adoption of educational technology.

In the realm of higher education, social influence was central to the adoption of educational technology. Specifically, social factors such as age, gender, field of study, ethnicity, and culture were critical in the blended learning environments of Malaysian higher education. These factors significantly affected students’ cognitive, emotional, and behavioural engagement, thereby influencing their level of participation in blended learning models [68]. Furthermore, research on the acceptance of AI-driven educational tools such as ChatGPT among Spanish university students showed that positive peer and social environment feedback significantly promoted the adoption of technology [69]. These studies highlighted the importance of social influence in the process of accepting educational technology.

In Pakistan, although social media was viewed as an important platform for enhancing knowledge in academic learning, its acceptance was still constrained by social perceptions and cultural differences [70]. In Bosnia and Herzegovina, during the COVID-19 pandemic, students’ preferences for online learning models were influenced by social factors such as field of study, year of study, and university status, demonstrating that social influence played a decisive role in the acceptance of educational technology [71]. Additionally, research indicated that the tendency to use smart devices was also directly influenced by sociodemographic variables, reflecting the critical role of social factors in the decision-making process of adopting educational technology [72].

These findings underscore the importance for educational decision-makers and technology developers to design and promote educational technologies that were better aligned with the specific social and cultural needs of their target users. This alignment was crucial in determining whether a technology could have been effectively adopted and implemented across different educational environments.

### Facilitating conditions

In today’s rapidly evolving educational environment, facilitating conditions became a crucial support for the implementation and adoption of new technologies in higher education. These conditions encompassed not only the availability and reliability of the technology itself but also the organisational and technical support provided by educational institutions. In the context of higher education, facilitating conditions pertained not just to the availability of technology but also to the effective implementation and widespread adoption of educational technologies.

Research investigating the adoption of educational technology products by university students identified key factors influencing their adoption, including technological infrastructure, ease of use, perceived usefulness, compatibility with existing academic practices, institutional support, and socio-cultural factors [18]. Specifically, a study in Spain found that although many professors use mobile devices in the classroom, concerns about potential distractions inhibited the integration and development of good teaching practices [73]. In China, during the COVID-19 pandemic, the use of mobile learning applications surged, but the effectiveness of these systems and student satisfaction was determined by the quality of institutional support and resource allocation [74]. Moreover, research at Central China Normal University highlighted the importance of optimising physical and resource spaces, emphasising the role of perceived infrastructure and resource modules in the construction of smart learning environments [75].

In terms of technical support and resource allocation, the emergency remote learning (ERL) experiences provided to higher education learners revealed that teaching effectiveness, interaction, and the quality of resources are key factors influencing students’ adoption of educational technology platforms [76]. Additionally, a study on the adoption of virtual communication technologies by educational leaders in India showed that facilitating conditions were among the primary factors influencing their willingness to adopt and actual use of these technologies [77].

Furthermore, research on professional teacher education methods demonstrated that introducing information technology and improving the planning, organisation, management, control, and quality of the educational process was crucial for enhancing the quality and personalisation of higher vocational education, highlighting the necessary level of education in an information-driven environment [78]. A survey of student educational preferences revealed that independence and flexibility in terms of time and location were particularly important to students, providing new insights into measuring student preferences for using virtual education platforms [79].

In summary, from technical support to the formulation of educational policies, facilitating conditions play a vital role in the adoption and expansion of educational technologies within higher education institutions. Continuous investment and strategic planning were essential in driving the sustained development and effective utilisation of educational technology.

## Conclusion

This study, through a systematic literature review, examined four key dimensions influencing the adoption of educational technology in higher education: Performance Expectancy, Effort Expectancy, Social Influence, and Facilitating Conditions. The findings indicated that technology adoption was a multi-faceted process shaped by individual, social, and organisational factors, which interacted to determine the effectiveness and sustainability of technology use. A holistic approach that considered these dimensions was essential for successful implementation.

Performance Expectancy influenced adoption by demonstrating how technology enhanced learning outcomes, while Effort Expectancy emphasised ease of use as a key factor for continued engagement [35]. Social Influence reflected the role of cultural contexts, peer support, and institutional policies in shaping technology acceptance [80]. Meanwhile, Facilitating Conditions, including infrastructure and institutional support, were crucial for sustained technology use.

These dimensions were interdependent rather than isolated. For instance, enhancing performance expectancy often required strong facilitating conditions, such as training and infrastructure. Likewise, improved ease of use (lower effort expectancy) strengthened social influence by encouraging adoption. Institutional endorsement of technology also shaped users’ perceptions of its value, reinforcing performance expectancy.

This study provided practical insights for higher education institutions to optimise technology adoption strategies and improve teaching outcomes. Future research should further investigate the complex relationships among these dimensions to develop a more comprehensive model for effective and equitable technology integration.

## Limitations and future directions

This study had several limitations. First, it relied primarily on published journal articles, excluding grey literature such as police reports, white papers, and dissertations, which could have provided additional practical insights. Second, the selection of databases (Scopus, WoS, and Emerald) may have introduced regional and linguistic biases, as non-English studies and region-specific publications were not included. Additionally, external factors such as policy changes, economic conditions, and institutional governance were not systematically analysed despite their potential influence on educational technology adoption.

Future research should address these limitations by expanding data sources to include grey literature and non-English regional studies for a more comprehensive perspective. Methodologically, mixed-methods approaches, such as quantitative bibliometric analysis and qualitative meta-synthesis, could provide deeper insights. Longitudinal and regional comparative studies would further clarify evolving adoption patterns and contextual differences. By integrating diverse data and methodologies, future research could develop a more inclusive and holistic framework for understanding educational technology adoption.

## Supporting information

S1 Text Search Terms. (DOCX)
