# Supplementary material for: Key factors influencing educational technology adoption in higher education: A systematic review
Source: PLOS Digit Health. 2025 Apr 29;4(4):e0000764. doi: 10.1371/journal.pdig.0000764 (PMC12040101; doi:10.1371/journal.pdig.0000764)
Supplement: S1 Text — (DOCX) [file pdig.0000764.s001.docx]

**S1 Text - Search Terms**

| **Databases** | **Search strings** |
| --- | --- |
| **SCOPUS** | ( TITLE-ABS-KEY ( educational AND technology ) AND TITLE-ABS-KEY ( determining AND factor ) AND TITLE-ABS-KEY ( higher AND education ) ) AND ( LIMIT-TO ( PUBYEAR , 2015 ) OR LIMIT-TO ( PUBYEAR , 2016 ) OR LIMIT-TO ( PUBYEAR , 2017 ) OR LIMIT-TO ( PUBYEAR , 2018 ) OR LIMIT-TO ( PUBYEAR , 2019 ) OR LIMIT-TO ( PUBYEAR , 2020 ) OR LIMIT-TO ( PUBYEAR , 2021 ) OR LIMIT-TO ( PUBYEAR , 2022 ) OR LIMIT-TO ( PUBYEAR , 2023 ) OR LIMIT-TO ( PUBYEAR , 2024 ) ) AND ( LIMIT-TO ( DOCTYPE , "ar" ) ) AND ( LIMIT-TO ( LANGUAGE , "English" ) ) |
| **Web of Science** | educational technology (All Fields) and determining factor (All Fields) and field of higher education (All Fields) and 2024 or 2023 or 2022 or 2021 or 2020 or 2019 or 2018 or 2017 or 2016 or 2015 (Publication Years) and English (Languages) and Article (Document Types) |
| **Emerald** | (content-type: article) AND (Determining the factors)AND(educational technology adoption in the field of higher education)AND2024 or 2023 or 2022 or 2021 or 2020 or 2019 or 2018 or 2017 or 2016 or 2015 (Publication Years) AND English (Languages) |
